# Modelling Vaccination Strategies against Rift Valley Fever in Livestock in Kenya

**DOI:** 10.1371/journal.pntd.0005049

**Published:** 2016-12-14

**Authors:** John M. Gachohi, M. Kariuki Njenga, Philip Kitala, Bernard Bett

**Affiliations:** 1 School of Public Health, Jomo Kenyatta University of Agriculture and Technology, Juja, Kenya; 2 International Livestock Research Institute, Nairobi, Kenya; 3 Kenya Medical Research Institute (KEMRI), Nairobi, Kenya; 4 Faculty of Veterinary Medicine, University of Nairobi, Nairobi, Kenya; George Washington University School of Medicine and Health Sciences, UNITED STATES

## Abstract

**Background:**

The impacts of vaccination on the transmission of Rift Valley fever virus (RVFV) have not been evaluated. We have developed a RVFV transmission model comprising two hosts—cattle as a separate host and sheep and goats as one combined host (herein after referred to as sheep)—and two vectors—*Aedes* species (spp) and *Culex* spp—and used it to predict the impacts of: (1) reactive vaccination implemented at various levels of coverage at pre-determined time points, (2) targeted vaccination involving either of the two host species, and (3) a periodic vaccination implemented biannually or annually before an outbreak.

**Methodology/Principal Findings:**

The model comprises coupled vector and host modules where the dynamics of vectors and hosts are described using a system of difference equations. Vector populations are structured into egg, larva, pupa and adult stages and the latter stage is further categorized into three infection categories: susceptible, exposed and infectious mosquitoes. The survival rates of the immature stages (egg, larva and pupa) are dependent on rainfall densities extracted from the Tropical Rainfall Measuring Mission (TRMM) for a Rift Valley fever (RVF) endemic site in Kenya over a period of 1827 days. The host populations are structured into four age classes comprising young, weaners, yearlings and adults and four infection categories including susceptible, exposed, infectious, and immune categories. The model reproduces the 2006/2007 RVF outbreak reported in empirical surveys in the target area and other seasonal transmission events that are perceived to occur during the wet seasons. Mass reactive vaccination strategies greatly reduce the potential for a major outbreak. The results also suggest that the effectiveness of vaccination can be enhanced by increasing the vaccination coverage, targeting vaccination on cattle given that this species plays a major role in the transmission of the virus, and using both periodic and reactive vaccination strategies.

**Conclusion/Significance:**

Reactive vaccination can be effective in mitigating the impacts of RVF outbreaks but practically, it is not always possible to have this measure implemented satisfactorily due to the rapid onset and evolution of RVF epidemics. This analysis demonstrates that both periodic and reactive vaccination ought to be used strategically to effectively control the disease.

## Author Summary

Evaluation of the relative impacts of RVF vaccination has not been previously carried out. We present a model that simulates RVFV transmission between two livestock hosts (cattle as a separate host and sheep referring to both sheep and goats) and two mosquito species (*Aedes* and *Culex* species). We then apply the model to evaluate policy-relevant impacts of vaccinating (1) different proportions of animals at different times to the simulated outbreak, (2) either of the host species, and (3) different proportions of animals in a periodic biannual or annual vaccination preventative strategy. Vector population growth is dependent on rainfall extracted from the Tropical Rainfall Measuring Mission (TRMM) for an RVF endemic site in Kenya over a period of 1827 days. The model reproduces the 2006/2007 RVF outbreak reported in empirical surveys in the target area and other seasonal transmission events that occur during the wet seasons. Consistent with anecdotal evidence, mass livestock vaccination can greatly reduce the potential for a major outbreak. The model predicts that the effectiveness can be improved by increasing the proportion of vaccinated animals, targeting vaccination against cattle and strategically augmenting periodic preventative strategies with reactive strategies once a RVF outbreak is predicted.

## Introduction

Rift valley fever (RVF) is a mosquito-borne viral zoonosis that causes periodic outbreaks accompanied by low-level virus activity during inter-outbreak periods mainly in sub-Saharan Africa [1]. The disease mainly affects sheep and goats, cattle and camels [2]. Humans can be exposed following a bite from an infected mosquito and or through direct contact with tissues from infected animals [3]. The disease was initially reported in restricted regions in Africa but has progressively spread to almost the whole continent, the island of Madagascar and the Arabian Peninsula [1][4]. The disease outbreaks often occur when favourable environmental drivers such as elevated and widespread rainfall and flat topography that promotes flooding [5] develop in areas where there are susceptible hosts [2] and competent mosquito vectors [6] and predisposing socio-economic practices such as herd replacement patterns [7].

Climatic factors seem to play a more dominant role as almost all historical outbreaks have been associated with cyclical patterns of the El Niño/Southern Oscillation (ENSO) phenomenon, which results in elevated and widespread rainfall over the Greater Horn of Africa (GHA) [5]. The outbreaks are often associated with adverse public health and economic impacts [8][9][10][11] as well as social impacts. Specifically, on a macroeconomic scale, Rich and Wanyoike [11] estimated that the 2006/2007 RVF outbreak in Kenya generated losses of over Ksh 2.1 billion (US$32 million then) on the Kenyan economy. The continued occurrence and geographical spread of RVF outbreaks points toward the need to understand the dynamics of the outbreaks as well as explore the approaches to their control.

Following the 2006/2007 RVF outbreak in Kenya, a retrospective analyses of the implemented responses revealed systematic delays due to the failure of the relevant institutions to recognize risk factors, act on early warnings messages (until the initial human cases were confirmed approximately two months after cases were observed in livestock) [12], and identify appropriate interventions. Consequently, stakeholders and decision-makers from the GHA region developed a risk-based Decision Support Framework (DSF) [12] that could be used to guide responses to similar emergencies in the future [12]. Livestock vaccination is one of the measures that were identified in the framework given that it has a good potential to reduce the impacts of the disease in livestock, contamination of the environment and subsequent exposure to humans [13].

There are many challenges that affect successful utilization of vaccines in the management of RVF outbreaks. First, the inter-outbreak period of the disease (approximated at 3–7 years [14]) is much longer than the shelf life of the currently available vaccine (*Smithburn* vaccine; 4 years) [12]. This discourages vaccine manufacturers from maintaining large stocks of these products given the risk of losing a large proportion of them through expiry. Most of these vaccines are often manufactured on order, for example, when the risk of an outbreak heightens. Secondly, the heavy rains and flooding that characterizes the high risk periods limit access and hence the delivery of vaccines to the rural areas. Thirdly, livestock species that are highly susceptible to the disease and hence would benefit from vaccination (such as goats and sheep) have a high population turn-over rates, limiting the maintenance of herd immunity especially in the pastoral areas. These challenges indicate an urgent need for policies that can guide utilization of RVF vaccines.

Mathematical models for simulating RVF epidemics have been developed [15] [16] [17] [18]. However, most of them are not suitable for evaluating vaccination strategies because they do not incorporate (i) climate variability (mainly precipitation changes) which greatly influences the timing of vaccination and other reactive interventions, and (ii) livestock population dynamics which influence the duration of herd immunity. We develop a model comprising two hosts—cattle as a separate host and sheep and goats as one combined host—and two vectors—*Aedes* species (spp) and *Culex* spp. Consequently, the model incorporates these components and use it to address policy-relevant questions on the effectiveness of reactive and periodic vaccination strategies including: (1) How can various vaccination coverages (VCs) implemented at different times before an outbreak affect the size of an outbreak in livestock? (2) To what extent is it possible to reduce outbreak size in both livestock species by focusing vaccination on one species? (3) How can periodic vaccination be used together with reactive vaccination particularly in the high risk areas? We incorporate two hosts with the recognition that pathogens such as RVFV that can infect multiple host species have different dynamics than single-host pathogens. Faced with scarcity of host-specific transmission parameters, this study sets the stage for the understanding of pathogen transmission dynamics and cost-effective control of RVF in multihost disease systems.

## Materials and Methods

### Model description

In developing the model, we make the following assumptions:

1. One time step denotes a day. The model is implemented using difference equations.
2. The model is developed based on data and some of the knowledge that have been gathered from Ijara sub-county, Kenya. The area is an RVF endemic site and was one of the epicenters during the last two outbreaks (1997/1998 and 2006/2007). Rainfall data used in the model were extracted from Tropical Rainfall Measuring Mission (TRMM) [19] based on the GPS coordinates for 17 high risk sites in the area for the period June 2006 to June 2007 to include the outbreak period between November 2006 and April 2007.
3. The ratio of cattle to sheep is 1:2. This is based on livestock census data collected in 2012 which estimated the populations of cattle, sheep and goats at 352,617, 323,676 and 348,648 respectively in the target area (District Veterinary office, annual report, 2012) Sheep and goat populations are combined and represented as sheep. In the model, these values have been scaled down to 6000 sheep and 3000 cattle. Hosts are classified into four age groups (young, weaner, yearling and adult groups) while vectors are classified into eggs, larvae, pupae and adults. The initial disaggregated number of sheep according to the respective age groups was young: 730, weaners: 840, yearlings: 1440, and adults: 2990. The initial disaggregated number of cattle according to the respective age groups was young: 210, weaners: 330, yearlings: 720, and adults: 1740. This age allocation was as per population structures obtained from empirical data collected during a participatory epidemiology survey in the study site. In modelling the population dynamics, hosts and vectors are subject to constant daily mortality rates.
4. RVFV transmission is thought to involve primary and secondary vectors. Primary vectors, which mainly comprise of floodwater *Aedes mcinthoshi*, are believed to act as reservoirs for RVFV as infected mosquitoes can transmit the virus trans-ovarially. Trans-ovarial transmission of the virus in infected *Aedes* species ensures that a proportion of mosquitoes emerges as infected adults and can, therefore, initiate transmission in livestock as they take their blood meals. Secondary vectors, on the other hand, include *Culex* species, *Mansonia* species, other mosquito species and experimentally, certain biting flies including phlebotomine sandflies and ticks [2].
5. The secondary vectors lay their eggs directly on water, and therefore, require stagnant water bodies for breeding. Such breeding environments always develop in flat or shallow depressions following increased precipitation and persistent flooding. The secondary vectors become infected when they feed on infectious livestock. When large populations of susceptible livestock are available, RVFV transmission is amplified by the secondary vectors as they take their blood meals. The model tracks all these processes including the primary and secondary RVFV transmission events by *Aedes* spp and *Culex* spp, respectively. Trans-ovarial transmission of the virus in *Aedes* species is not modeled explicitly. In addition, *Culex* spp. is assumed to represent all the secondary vectors of RVFV.
6. In modelling mosquito infection dynamics, the vector population is divided into susceptible, exposed and infectious segments (S-E-I model). Susceptible vectors represent the proportion that can become infected if they ingest blood from an infectious host. Exposed vectors are infected with the virus but are not yet capable of transmitting the virus to a susceptible host until a latency period has elapsed. Infectious vectors are capable of transmitting the virus to a susceptible host and infectious vectors remain infected for life. Super infections are ignored.
7. In modelling host infection dynamics, the host population is divided into susceptible, exposed, infectious and recovered segments (S-E-I-R model). Susceptible hosts represent the proportion that can become infected if an infectious vector feeds on it. Exposed hosts are infected with the virus but are not yet capable of transmitting the virus for a defined period of time, i.e. the latent period. Infectious hosts are capable of transmitting the virus to a susceptible vector. Infectious hosts suffer an additional RVF-induced mortality but if they recover from the infection, they remain immune. Infectiousness is assumed to be similar during the infectious periods in hosts. Super infections in the hosts are also ignored.
8. Naturally, all livestock are susceptible to RVFV infection although there are differences in susceptibility across species and ages. In the model, the susceptibility to RVFV of the two host species considered is assumed to be similar. However, in parameterizing RVF-induced mortality, the case fatality rates for the young animals are higher than those of other age classes (weaners, yearlings and adults).
9. The duration of the latent and infectious periods in vectors is assumed to be similar in both vector species as well as between the host species.
10. Differences in body surface areas between cattle and sheep are accounted for blood feeding by mosquitoes. The surface area of cattle is 3m^2^ while that of sheep is 0.83m^2^.
11. The blood meal obtained from each host by each vector species is weighted using two parameters–the relative population of cattle and sheep to determine probability of a mosquito feeding on host and surface area of cattle and sheep exposed to bites. To compute blood meal index, the weighting is implemented as follows: Sheep: 6000*0.83 = 4980; Cattle: 3000*3 = 9000; Blood meal from sheep = 4980/(4980+9000) = 0.356. Blood meal from cattle = 9000/(4980+9000) = 0.644.

### The vector module

#### Aedes species population dynamics

Table 1 illustrates input parameters used for simulating *Aedes* species population dynamics. They include buried *Aedes* eggs hatching rate, larval development rate and daily mortality rate, pupal development rate and daily mortality rate and adult daily mortality rate. Equations used in this model are described in the Supplementary Text S1 Text. Briefly, the hatching rate of *Aedes* eggs is assumed to follow a fuzzy logic model that was developed and used by Emert et al. 2011 [20] to model population dynamics of anopheles mosquitoes. The principle exemplified by the fuzzy logic model is assumed to be relevant for *Aedes* spp because: (1) none or a small number of *Aedes* eggs hatch under little amounts of rainfall, (2) total inundation of breeding sites with water leads to a high hatching rate of the eggs, and (3) there is a sharp decline of adult numbers once extensive flooding occurs. During the flooding period, the breeding sites of *Aedes* spp become unsuitable from the washing effect of larvae and from the fact that the eggs hatch only when more than six days of dry conditions are present following oviposition to facilitate egg maturation. In addition, studies conducted by Linthicum et al. (1983) [21] on *Aedes* larvae collections showed that initial samples were collected on day 9 following a period of heavy precipitation. These collections peaked on days 12 and 13 but declined when extensive flooding persisted and the last collections were observed on day 21 [21]. The fuzzy model, therefore, distinguishes between dry unsuitable conditions (threshold ∪_*1*_), a most suitable condition (*S*), and unsuitable conditions due to very high rainfall and flooding (threshold ∪_*2*_). The model generates a distribution of values between 0 and 1 with high values denoting a good suitability of a habitat for the development of *Aedes* spp.

**Table 1:** Parameters table.

Parameter	Symbol	Value	Source
***Aedes* species population dynamics parameters**			
Buried *Aedes* eggs hatching rate	*h*_*A*_	0.33	[22]
*Aedes* larva daily mortality rate	*AdLμ*	0.2	[23]
*Aedes* pupa development rate	*Adlp*	0.2	[24]
*Aedes* pupa daily mortality rate	*AdPμ*	0.1	[25]
*Aedes* adult daily mortality rate	*AdAμ*	0.1	[26]
***Culex* species population dynamics parameters**			
*Culex* eggs hatching rate	*h*_*C*_	0.33	[27]
*Culex* eggs mortality rate	*CxEμ*	0.01	[28]
*Culex* larva daily mortality rate	*CxLμ*	0.2	Subjective estimate*
*Culex* larva development rate	*Cxlp*	0.1	[27]
*Culex* pupa development rate	*Cxpa*	0.2	[29]
*Culex* pupa daily mortality rate	*CxPμ*	0.1	Subjective estimate*
*Culex* adult daily mortality rate	*CxAμ*	0.09	[30]
Number of eggs laid per day by one mosquito	*S*_*C*_	40	[31]**
*Culex* eggs carrying capacity	CxECC	200,000	-
**Logistic regression model parameters used to grow *Culex* mosquito population**			
Logistic model constant	*β*_*o*_	-6.776691	-
Coefficient for the counter variable	*β*_2_	0.263765	-
Coefficient for the counter variable squared	*β*_2_^2^	-0.0022497	-
Daily value of the counter *variable*	*x*_*2*_	Daily value	-
**Host parameters**			
Cattle birth rate	*b*_*c*_	0.00275	-
Period (days) spent as a young calf	*δc*	150	-
Period (days) spent as a weaner cattle	*τc*	210	-
Period (days) spent as a yearling cattle	*ϕc*	550	-
Cattle mortality	*μC*	0.000611	-
Cattle carrying capacity	*CCC*	4000	-
Adult cattle offtake	*Ȱ*_*c*_	0.0001	-
Sheep birth rate	*b*_*c*_	0.005	-
Period (days) spent as a young lamb	*δs*	150	-
Period (days) spent as a weaner sheep	*τs*	210	-
Period (days) spent as a yearling sheep	*ϕs*	365	-
Sheep mortality	*μS*	0.000814	-
Sheep carrying capacity	*SCC*	7000	-
Adult sheep offtake	*Ȱ*_*s*_	0.0003	-
**Transmission-based parameters**			
Vector feeding rate	*Ƒ*	0.33	[32][33]
Host infectivity	*Ƕ*_*h*_	0.14	[18]
Proportion of *Culex* blood meals from cattle	*Ѫ*	0.5	-
*Aedes* species infectivity	*Ƕ*_*A*_	0.62	[34]
*Culex* species infectivity	*Ƕ*_*C*_	0.6	[34]
Latent period (days) in hosts	*ε*	3	[15][35]
Infectious period (days) in hosts	*γ*	6	[2][6]
Latent period (days) in vectors	*ε*_*v*_	3	[34]
RVF-specific mortality in calves	*σ*_*CC*_	0.4	[2]
RVF-specific mortality in other cattle	*σ*_*AC*_	0.075	[2]
RVF-specific mortality in lambs	*σ*_*LS*_	0.95	[2]
RVF-specific mortality in other sheep	*σ*_*AS*_	0.2	[2]

*similar to *Aedes* species;

**An adult female *Culex* lays between 200 and 300 eggs every 3 days, so we assumed an average lay 80 eggs per day. Assuming a sex ratio of 1:1, and because only females are modelled, we end up with 40 eggs laid per day

To improve the predictive ability of the model, various combinations of cumulative rainfall (specifically at 3, 7, 14 and 21 days) and threshold parameters for the fuzzy model were analyzed. A 21-day cumulative rainfall (*R*Σ*21d*) with ∪_*1*_ = 0, *S* = 5mm and ∪_*2*_ = 8mm generated a reasonable prediction that did not only capture variability in the *Aedes* mosquito population over time but also allowed for a smooth transition in the vector densities from the primary to secondary (*Culex* species) RVFV vectors. Details of how the fuzzy suitability (*f*) of *R*Σ*21d* is computed and implemented in the hatching of *Aedes* eggs are in the Supplementary Text S1 Text.

#### Culex species population dynamics

Published data indicate that the population densities of *Culex* spp and other secondary vectors of RVFV (*Mansonia* spp, *Anopheles* spp, etc) increase tremendously when precipitation persists for at least 28–42 days [21]. Although this process is thought to be one of the key determinants of an RVF outbreak, no information exists on the amount of rainfall or minimum duration of flooding that would be required to enable the development of these vectors to critical population densities. To address this challenge, we studied the distribution of rainfall (TRMM) in the 17 sites in the study area where the last RVF outbreak (November 2006 –April 2007) occurred over a one year period (June 2006 to June 2007) and used a logistic regression model to identify a pattern that could be associated with the outbreak. A new rainfall-associated variable was generated from the daily rainfall that included running cumulative number of wet days, where a wet day was when the cumulative rain over 28 days exceeded 2, 4, 6, 8 or 10 mm. The 28 day-cumulative rainfall was used as a proxy for heavy precipitation and flooding while the number of wet days controlled for persistence or longevity of precipitation.

A dummy variable indicating presence or absence of an outbreak on day *i* was derived and used as an outcome variable in a logistic regression model to identify the number of wet days that gave the best fitting model based on deviance statistics. The model selected was used to generate probabilities representing the likelihood of an outbreak occurring based on the changes in precipitation levels. The model was then used to generate a probability distribution that could be used to control the population dynamics of *Culex* mosquitoes assuming changes in the densities of these vectors has a direct influence on the risk of an outbreak. The parameters of the regression model generated are described in Table 1 and the details on how the fitted values were incorporated into the *Culex* mosquito’s population dynamics model are illustrated in the Supplementary Text S1 Text.

#### The livestock module

The livestock module simulates population dynamics of each livestock species considered as well as the rates of transmission of the virus in the population, assuming that each host has an equal chance of being bitten by an infectious vector. Host populations are classified into young, weaner, yearling and adult compartments. For both species, these compartments correspond to the age limits of: ≤ 5 months, >5 months and ≤1 year, >1 and <2 years and > 2 years, respectively. Cattle give birth to susceptible young animals at a per capita rate of *b*_*c*_; they mature to weaners at a per capita rate of 1/*δc*. Weaners develop to yearlings at a per capita rate of 1/*τc* while yearlings develop to adults at a per capita rate of 1/*ϕc*; these parameters are described further in Table 1. All these age categories experience a baseline mortality rate of *μC* but adults also exit the population through an offtake at a rate of *Ȱc*. Similarly, sheep give birth to young animals at a per capita rate of *bs*; they mature to weaners at a per capita rate of 1/*δs*. Weaners transit to yearling sheep at a rate of 1/*τs* and yearlings mature to adults at a rate of 1/*ϕs*. All the sheep age categories suffer a baseline mortality rate of *μS* and adult sheep are further removed from the population through an offtake rate of *Ȱs*.

#### RVFV transmission

**Fig 1:**
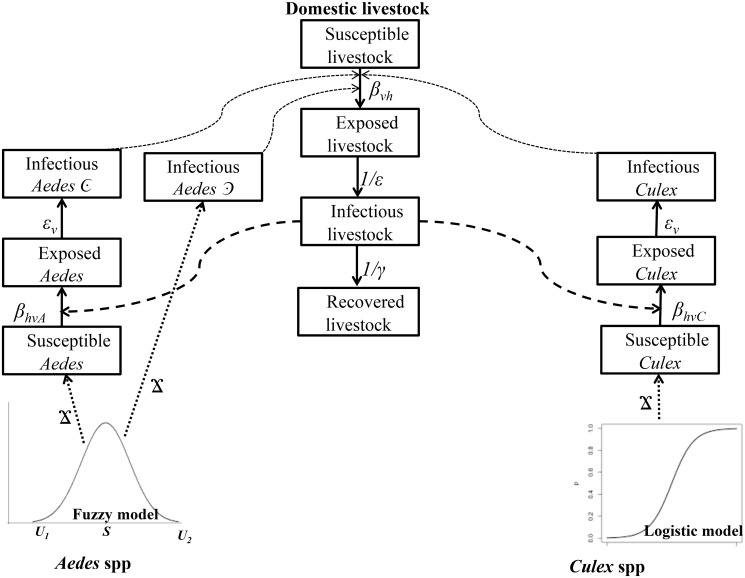
Summary flow diagram of the model structure demonstrating the bidirectional RVFV transmission between domestic livestock and the two mosquito species. The structure also shows the infection states of livestock and mosquitoes and the parameters that describe transition pathways. *Aedes* spp population growth is governed by a fuzzy distribution model that leads to development, through explicitly modelled aquatic stages (Ϫ), of either susceptible or infectious adults. *Culex* spp population growth is governed by a logistic distribution model that leads to development, through explicitly modelled aquatic stages (Ϫ), of susceptible adults. See vector aquatic stages modelling in the text.

Functions used to analyze the transmission of the RVFV between hosts and vectors are adapted from Smith et al. 2012 [36]. A schematic representation of these processes is demonstrated in Fig 1.

#### Hosts

The rate of transmission of the virus from an infectious vector to a susceptible host is represented by *β*_*vh*_ (*β*_*vh*_*C* for cattle and *β*_*vh*_*S* for sheep). *β*_*vh*_ is a composite variable obtained by multiplying:

1. *Vector biting rate*, *Ƒ*, estimated as an inverse of the vector’s gonotrophic interval; this is assumed to be equal for both vectors (Table 1)
2. *Host infectivity*, *Ƕ*_*h*_, the probability that a bite by an infected mosquito results in an infection in a susceptible host. The value of this parameter is also assumed to be equal across all ages and species (Table 1)
3. *Vector*: *host ratio*: this is computed on a daily basis for each vector and host species by dividing the population of a given vector species by a given host species. The parameters used in this case are *ʤAC* representing *Aedes*:cattle ratio; *ʤAS* for *Aedes*: sheep ratio; *ʤCC for Culex*: cattle ratio and *ʤCS* for *Culex*: sheep ratio.
4. *Vector blood meal index*, *Ѫ*, is a measure of the proportion of blood meals obtained by a given vector species from a given host species. It is assumed that each vector has equal chances of biting any of the two hosts (Table 1)
5. *RVFV prevalence in the vector* is an output generated by the model on a daily basis; *prA* is the RVFV prevalence in *Aedes* mosquitoes and *prC* is the RVFV prevalence in *Culex* mosquitoes.

A composite value of the force of infection (FoI) on a given host is obtained by adding up the vector-specific FoI estimates as follows:

$$\beta_{vh}C=(Ƒ(Aedes)*{Ƕ}_{h}*ʤAC*\;Ѫ(Aedes)*prA)+(Ƒ(Culex)*{Ƕ}_{h}*ʤCC*\;Ѫ(Culex)*prC)$$

$$\beta_{vh}S=(Ƒ(Aedes)*{Ƕ}_{h}*ʤAS*\;Ѫ(Aedes)*prA)+(Ƒ(Culex)*{Ƕ}_{h}*ʤCS*Ѫ(Culex)*prC)$$

To track hosts’ infection dynamics, each of the age categories presented above are further classified into four additional states: susceptible, exposed, infectious and recovered categories. The rate of transition from a susceptible to exposed category is determined by the force of infection. Exposed animals transit to the infectious state at a per capita rate of 1/*ε*, while infectious livestock transit to the recovered state at a rate 1/*γ*. Infectious young and other cattle suffer an additional, disease specific mortality (case fatality rate) of *σ*_*CC*_ and *σ*_*AC*_ (Table 1). Infectious young and other sheep suffer an additional, disease specific mortality of *σ*_*LS*_ and *σ*_*AS*_ (Table 1). Hosts that recover from the infection remain immune for life.

#### Vectors

Similarly, the adult stages of mosquitoes are reclassified into susceptible, exposed and infectious categories depending on their infection status. The rate at which a susceptible vector transits to exposed state is governed by vector-specific FoI, *β*_*hv*_ which is computed based on:

1. *Vector biting rate*, *Ƒ*, estimated as an inverse of the vector feeding interval
2. *Vector infectivity* i.e. probability that a bite on an infected host results in an infection in the vector— *Ƕ*_*A*_ for *Aedes* and *Ƕ*_*C*_ for *Culex*
3. *Vector blood meal index*, *Ѫ*,
4. *RVFV prevalence in the hosts*: this is generated by the model on daily basis based on the input parameters offered to the model. The symbols for these estimates are: *prCatt* for cattle and *prShp* for sheep.

The composite vector-specific FoI is the sum of the components derived from each of the livestock species as follows:

$$\beta v_{hA}=(Ƒ(Aedes)*{Ƕ}_{A}*\;Ѫ(Aedes)*prCatt)+(Ƒ(Aedes)*{Ƕ}_{A}*Ѫ(Aedes)*prShp)$$

$$\beta v_{hC}=(Ƒ(Culex)*{Ƕ}_{C}*\;Ѫ(Culex)*prCatt)+(Ƒ(Culex)*{Ƕ}_{C}*Ѫ(Culex)*prShp)$$

The rate at which an exposed mosquito transits to an infectious state is given by 1/*ε*_*v*_ where *ε*_*v*_ is the latent period of the virus in the vector. Infected vectors remain so for life. A system of difference equations used for this model is presented in the Supplementary Text S1 Text.

### Model analyses

#### Simulation

The host population model is run for twenty two years in order to attain a stable equilibrium. After this, RVFV infections are introduced with outputs generated being (1) time to the peak incidence of RVFV, (2) duration of the outbreaks, and (3) cumulative incidence in both vectors and hosts.

#### Sensitivity analyses

Sensitivity analysis was carried out to determine the relative importance of the model parameters with respect to RVFV cumulative incidence. This was done by varying the baseline values of each parameter in turns by ±50%. The effects of the changes made were assessed by determining proportional (%) change in cumulative incidence from the baseline level. Changes in the values of the most sensitive parameters were expected to result in substantial impacts on the cumulative incidence.

#### Scenario analyses

Three vaccination scenarios were analyzed using the model. These included: (i) reactive vaccination implemented at various time points before the outbreak, (ii) periodic vaccination implemented over a two-year period at six-monthly intervals and (iii) vaccinating one of the two host species. For the first two scenarios, levels of vaccination coverage are varied from 5 to 100% at intervals of 5%. The impacts of all the interventions evaluated are assessed by determining the proportional reduction in cumulative incidence. Key assumptions made for the analysis are:

1. It takes 7 days for vaccinated animals to become protected following vaccination
2. Vaccines have an efficacy of 50–100% in both hosts
3. Vaccinations are administered efficiently with no wastage
4. The vaccine being used has no deleterious effects on the host

In most mathematical models, vaccination is often implemented as a pulse event depending on time, level of coverage and interval of vaccination desired. However, this approach does not reflect vaccination patterns observed in the field because it usually takes days to weeks of continuous vaccination, depending on the number of vaccinators deployed, to attain the coverage required. In an attempt to mimic observed patterns, a constant number of animals are transferred from the susceptible to vaccinated on daily basis until the target vaccination coverage is achieved. The number of animals moved per day is determined based on vaccination data obtained from the target area which suggest that one technician can vaccinate 1000 cattle or 2000 sheep in a day and that two teams, each comprising of 16 technicians are often deployed in such campaigns. It would, for example, take 5 days, 11 days and 15 days to achieve 25%, 50% and 75% coverage in the area.

#### Reactive vaccination implemented at successive time points before the outbreak

Up to 13 vaccination time points at two-weekly intervals starting at 24 weeks to the onset of the outbreak were used in this scenario. These times points include those that have been identified in the RVF Decision Support Framework that was developed to guide the implementation of RVF interventions, including vaccination [12].

#### Impact of periodic vaccination strategies

Periodic vaccination strategies assessed included biannual and annual vaccinations over a period of 2 years prior to the outbreak.

#### Impact of vaccinating either of the two host species

This analysis assesses the impact of targeting one of the two host species for vaccination. 50% VC was implemented in either of the hosts at the onset of the outbreak.

#### Ethics statement

There was no direct involvement of either human or animal subjects in this study. Therefore, the study protocol did not require institutional review board approval.

## Results

### Simulation of mosquito population dynamics

**Fig 2:**
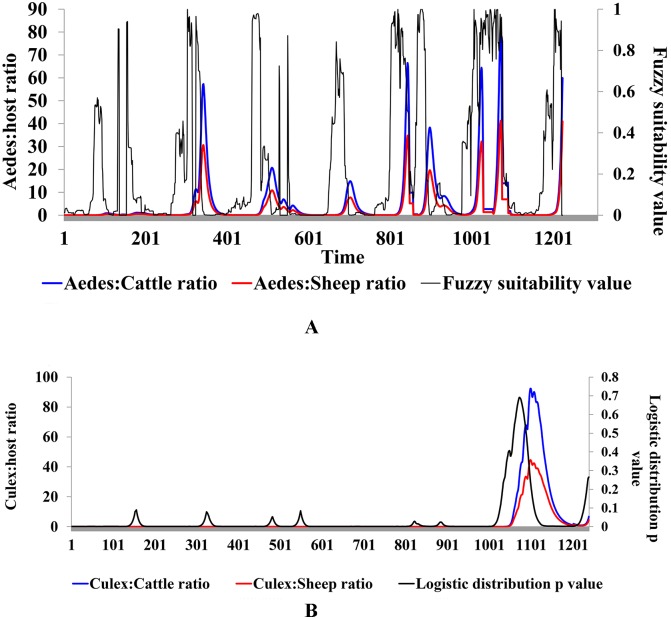
Predicted temporal relationship between fuzzy (panel A) and logistic probability functions (panel B) and vector: host ratios.

Two probability distributions generated using the fuzzy and logistic regression models based on TRMM rainfall values are successfully used to drive *Aedes* and *Culex* mosquito populations, respectively. Fig 2 shows the temporal relationship between these probability distributions and the respective vector:host ratios. In general, peaks in vector:host ratios lag those of fuzzy and logistic probability distributions by approximately 8–17 days and 30 days, respectively. Between days 9256 and 9450 when there was heavy/persistent rainfall, the fuzzy and logistic regression models generated high probability values which led to an upsurge in the mosquito populations, hence high vector:host ratios (Fig 2). The other wet seasons before this had short-lived precipitation events that were not adequate to support an upsurge of the *Culex* mosquito population though that of *Aedes* mosquitoes responded positively.

### Infection dynamics in vectors and hosts

In the simulated outbreak, *Aedes* adults that emerge from infected eggs, last for a total of 148 days and peak at day 80. Susceptible *Aedes* mosquitoes also develop at the same time peaking on day 87. *Culex* mosquito population appears 36 days after the emergence of *Aedes* population. *Culex* mosquitoes gain RVFV infection from viraemic hosts from day 69 after initial transmissions by *Aedes* spp. The maximum FoI exerted to *Aedes* spp from cattle and sheep are 0.016 and 0.006 respectively. The maximum FoI exerted to *Culex* spp from cattle and sheep are 0.015 and 0.0057 respectively.

**Fig 3:**
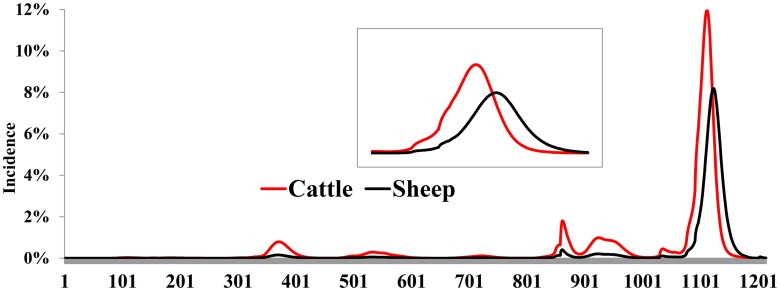
Simulated incidence of RVFV in hosts over 1200 days. The inset graph is a magnification of the full-blown outbreak period.

Predicted RVFV incidence in hosts is shown in Fig 3. These predictions show five transient RVFV transmissions associated with seasonal rains and one main outbreak associated with heavy and persistent precipitation. In general, seasonal transmission events fail to result in full-blown outbreaks given that no amplification of populations of *Culex* spp occurs (Fig 2). The outbreak curve has a characteristic shape–RVFV activity begins slowly until *Culex* spp population surges, resulting in the amplification of the virus. The predicted peak outbreak incidence of RVFV in cattle is 12% on day 112 of the outbreak while that for sheep is 8% on day 123. The predicted duration of the outbreak is 184 days. The maximum force of infection exerted to cattle and sheep are 0.24 and 0.06, respectively.

### Scenario analyses

A simulated RVF outbreak in this study was defined by noting the predicted peak endemic incidence in hosts. The peak endemic incidence was used as the threshold for definition of an outbreak. By comparing endemic verses epidemic patterns predicted in the model, it appears that the number of cases predicted during the outbreak captured is 80% more than those predicted for the endemic periods. We use the 80% threshold for evaluating impacts of the various vaccination scenarios being studied.

#### Impact of reactive vaccination at different times to RVF outbreak

**Fig 4:**
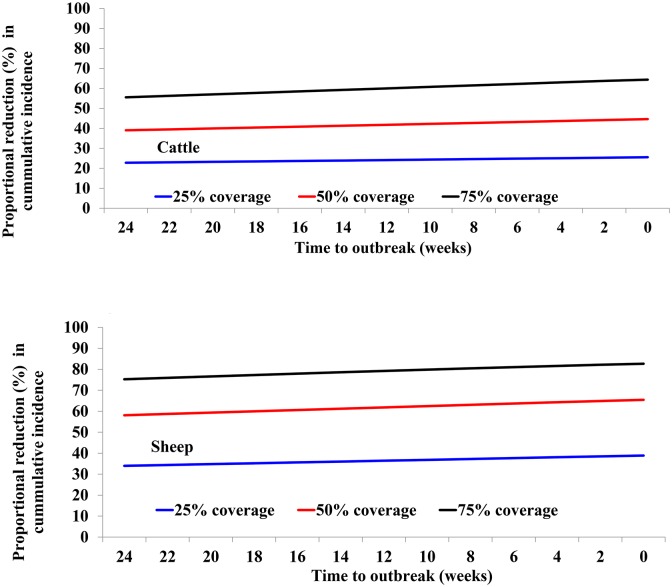
Estimated proportion of cases averted for different vaccination coverages and at different times to the outbreak in cattle (top panel) and sheep (bottom panel).

Fig 4 shows the impacts of reactive vaccination for three levels of coverage: 25%, 50% and 75% that are used for demonstration. The main observations from this analysis are: (i) a low VC of 25% can achieve a reduction in RVFV incidence of at least 22% in each host species, (ii) vaccination would have more impacts on RVFV incidence in sheep than cattle, (iii) the higher the level of VC, the higher the proportional reduction in the RVFV cumulative incidence, and (iv) varying the timing of the vaccination between 0 and 24 weeks results in changes in the impact of vaccination. For example, vaccinating 25%, 50% and 75% of hosts at the onset of the outbreak compared to 24 weeks earlier results in only 3%, 5% and 9% reduction in the cumulative incidence of RVFV in cattle and 5%, 7% and 7% in sheep, respectively.

**Fig 5:**
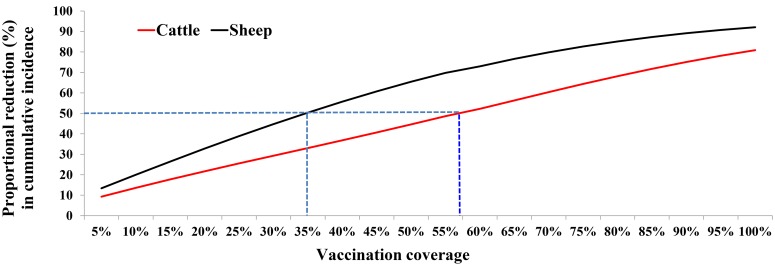
Estimated proportion of cases averted for different vaccination coverages implemented at the onset of outbreak. The dashed blue line shows the predicted vaccination coverage estimated to reduce the cumulative incidence in each host species by 50% in an outbreak.

An additional analysis that focusses on the relationship between herd immunity at the start of the outbreak and RVFV cumulative incidence (Fig 5) show that a herd immunity of approximately 35% in sheep and 60% in cattle will be needed to avert 50% of the cases in each host population.

#### Impact of a periodic vaccination strategy

**Fig 6:**
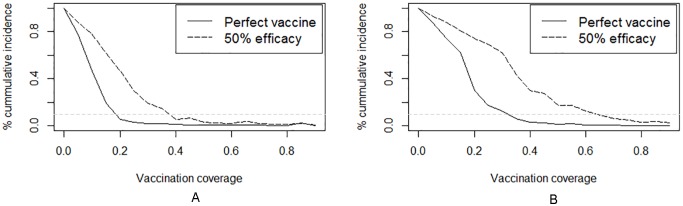
Expected impacts of biannual (Panel A) and annual (Panel B) periodic vaccination scenarios on the cumulative incidence of RVFV using a perfect vaccine and a vaccine with 50% efficacy.

Fig 6 shows expected impacts of biannual and annual periodic vaccination scenarios on the cumulative incidence of RVFV using a perfect vaccine and a vaccine with 50% efficacy. The results suggest that periodic vaccination results in a progressive enhancement of the herd immunity with time, more so in biannual than annual vaccination strategies. In this scenario, we use the 90% threshold for evaluating vaccination impacts (refer to Fig 6 that illustrates the threshold (horizontal) line to show that VC above the line are not effective in stopping the outbreak). For the biannual vaccination, the impacts of the intervention implemented over a two year period are >90% at a VC of 20% with a perfect vaccine and a VC of 40% with an imperfect vaccine. In an annual vaccination regime, similar levels of impacts (>90%) would be achieved with a VC of approximately 30% with a perfect vaccine and a VC of approximately 65% with an imperfect vaccine.

**Fig 7:**
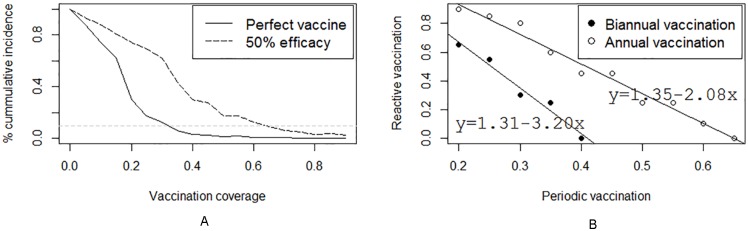
Impacts of integrating various levels of routine and reactive vaccination required to stop an RVF outbreak using a prefect vaccine (Panel A) and imperfect vaccine with 50% vaccine efficacy (Panel B).

Fig 7 shows impacts of integrating reactive VCs (refer to Fig 4) with periodic VCs (refer to Fig 6). Equations are given for each scenario that can be used to determine the level of reactive vaccination that will be required to stop an outbreak, given a specific level of periodic vaccination implemented.

#### Impact of targeting vaccination against either of one host species

**Fig 8:**
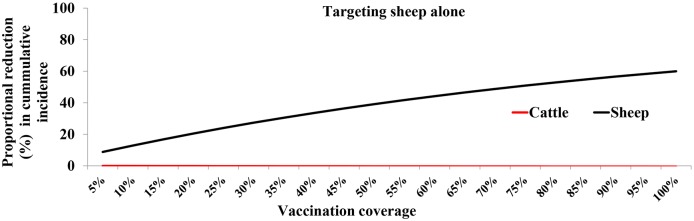
Estimated proportion of cases averted for different vaccination coverages implemented at the onset of outbreak by either targeting cattle (top panel) or sheep (bottom panel) alone.

Vaccinating cattle alone in the population at simulated VCs confers protection to both cattle and sheep (Fig 8). On the other hand targeting sheep alone confers protection to sheep population only (Fig 8).

### Sensitivity analyses

Sensitivity analyses showed that the infectious period and infectivity in both hosts and vectors (particularly *Culex* spp) were sensitive to the cumulative incidence of RVF. Others included survival and mosquito biting behavior of *Culex* spp (Table 2).

**Table 2:** Proportional (%) change in cumulative incidence in cattle and sheep upon a ±50% of model parameter values.

Parameter	-50%		+50%	
	Cattle	Sheep	Cattle	Sheep
Livestock infectivity	9	40	57	37
*Aedes* adult mortality	84	68	4	4
*Culex* spp infectivity	58	80	25	4
*Culex* spp biting rate	41	67	25	1
*Culex* spp larval mortality	37	62	53	77
*Culex spp* pupa development	66	85	24	1
*Culex* spp adult mortality	23	7	53	77
*Culex* spp pupa development	95	97	5	16
Both vectors spp adult mortality	9	30	63	75
Both livestock sppp infectious period	60	84	55	18
Cattle alone infectious period	48	77	59	22

## Discussion

### The model

We present a deterministic model that combines precipitation patterns, mosquito population dynamics and host demographics to simulate RVFV transmission. The model predicts elevated RVFV activity during the wet seasons as well as a full-blown RVF outbreak following periods with excessive and persistent precipitation. Elevated and persistent rainfall is a risk factor for RVF outbreaks—all the 11 reported RVF outbreaks in Kenya occurred in years when the average annual rainfall increased by more than 50% in the affected districts [14].

The novelty of the model is in the bridging of separate probability distributions that uses satellite-derived daily precipitation for the study area that ensure temporal succession of separate vector species population growths. Since we are not interested in the importance of trans-ovarial transmission and its implications on the generation of the outbreak [17], we exclude these detailed dynamics in *Aedes* mosquitoes. Adult *Aedes* mosquito emergence events are dependent on water (rainfall) that inundates breeding habitats [21]. We, therefore, base the dynamic distribution of *Aedes* species on accumulated rainfall amounts using a fuzzy distribution model similar to that employed by Emert et al. (2011) [20]. The fuzzy distribution model computes dynamic suitability conditions of hatching of *Aedes* eggs that mimic the reported strong relationship between *Aedes* mosquito emergence and weather (rainfall) variability [21]. The assumptions driving the fuzzy distribution model, as described in the *Methodology* section, seem rational and might denote a qualitatively plausible relationship of *Aedes* egg hatching process than a simple linear function of rainfall.

*Culex* mosquito population dynamics are driven using an approach of obtaining parameters from a statistical analyses of reports of livestock cases and a particular pattern of rainfall during the 2006/2007 outbreak. We used this function based on empirical studies that reported that the mosquito breeding sites were colonized by massive swarms of *Culex* (and other species) if they remained flooded for at least 28–42 days [21]. Additionally, livestock keepers in the study area reported a mean average of 23 days between the start of heavy rains and the appearance of mosquito swarms during the 2006/2007 RVF outbreak [8], though most likely these included both primary and the secondary species. Our model accurately captures this temporal relationship between cumulative rainfall and secondary mosquito species emergence.

A different approach of growing seasonal vector populations in modelling RVFV transmission in West Africa was implemented by Soti et al. [37] using a hydrology model. Their hydrological model uses daily rainfall as input to simulate variations of water pool surface areas. We have not used this approach as the epidemiology of RVF occurrence in West Africa and GHA is different. Whereas in GHA RVF outbreaks are known to be closely associated with ENSO phenomenon [5], periods of RVF outbreaks in West Africa do not necessarily coincide with years of highest total rainfall [38]. Indeed, RVF epidemiological landscape in West Africa is influenced by the generation of temporary ponds and a particular rainfall temporal distribution (populations of *Aedes* and *Culex* spp depend on the alternation of rainy and dry periods) [38]. Although rainfall, just as in GHA, is the main driver of hydrologic dynamics of water pools in West Africa, the mechanistic vector productivity of specific habitats and RVFV transmission and the consequent epidemiological inference in the two ecologies can be substantially different. Empirical studies are needed in the two distinct ecologies to accurately quantify the amount and distribution of rainfall regimes (and how they interact with soil infiltration rates) required for hatching of primary vectors.

We implement the legendary assumption which considers primary and secondary vectors playing a synergistic role in generation of RVF outbreaks. Innovative ways of empirically examining these assumptions are needed to answer questions such as whether primary vectors alone [17] or whether secondary vectors alone (for example, if augmented with movements of animals) [7] can drive RVF full-blown outbreaks. In addition, this model hypothesizes that water availability may play a more dominant role in driving the vectors population dynamics. Future model refinements should incorporate not only the effects of temperature and humidity, vegetation and nutrient competition on vector population dynamics but also on the extrinsic incubation periods of RVFV in vectors [39].

### Modelling vaccination strategies

For RVF control to be evaluated and optimum control strategies devised, an increased understanding of the transmission dynamics among hosts and vectors is paramount. In this way, we apply the model to identify the key factors driving the number of potentially averted RVF cases in a simulated outbreak. The analyses show that vaccination, as a sole intervention, can be effective in mitigating the impacts of RVF outbreaks. The success of RVF vaccination is predicted to be defined by the targeted vaccination coverage and the time to the outbreak. The proportion of cases averted is related to the targeted vaccination coverage, particularly for low levels. The policy implication of this prediction is that resources and planning required to achieve a given VC corresponds to the number of cases expected to be averted. For a given VC, higher herd immunity at the outbreak onset is predictably highly beneficial. Vaccinating early reduces herd immunity, over time, through removal of immune animals via expected mortality and offtake and birth of susceptible animals. The model predicts that 3–6% more cases can be averted if, for the simulated VCs, vaccination is implemented close to the outbreak. Averting 3–9% more cases can lead to large numbers of deaths being averted particularly in the more RVF-induced mortality susceptible species such as sheep. For greater effectiveness, this prediction implies that a careful balance between a given VC and optimal timing is critical. These predictions concur with recent modelling study predictions that a higher rate of vaccination may help to reduce the epidemic size and a maximal attempt of vaccination just before an outbreak is highly beneficial [18]. In sub-Saharan Africa, vaccination against RVFV has been used for many years either to prevent disease occurrence [40] or to mitigate disease impacts [41]. Our model predictions clearly demonstrate the usefulness of effective implementation of this intervention. Ideally, however, all members of a population need not be vaccinated because as the number of susceptible hosts in the population is reduced, the efficiency with which a pathogen is transmitted is greatly reduced (the concept of herd immunity) [42]. The model predicts that this indirect protection is accelerated as vaccination coverage is increased and, moreover, it is experienced more in sheep relative to cattle.

Early and optimal timing, in turn, depends upon a sensitive and functioning RVF surveillance and prediction system and a rapid response capacity by the national veterinary authorities [40]. One such surveillance system integrates ENSO related climate anomalies including elevated sea-surface temperatures and satellite-derived normalized difference vegetation index data (NDVI) [5]. During the 2006/2007RVF outbreak, this system retrospectively provided a 2 to 4 month period of warning in the GHA region [5]. However, the RVF DSF estimates the lead-time to order, produce, deliver sufficient vaccine to the field and attain herd immunity in livestock to be approximately 5 months [12]. This implies that vaccine orders need to be placed prior to the first RVF early warning. Currently, this is impractical unless the lead time for prospective predictions of RVF outbreaks is lengthened. Still, even if the latter were achieved (to, e.g. 5 months), mobilizing adequate resources to procure the vaccines within the short period is a difficult task in resource-scarce countries in the GHA. Moreover, by this time, the co-occurrence of heavy rains and flooding in the rural areas coupled with the absence of all-weather roads can present huge logistical challenges in vaccine delivery. Innovative strategies are clearly needed as part of outbreak preparedness plan.

To overcome some of these challenges, the RVF DSF proposes a strategic regional vaccine shared bank which could be rapidly deployed in times of need [12]. To supplement this proposition, we modeled a periodic vaccination strategy implemented under different vaccination coverage biannually or annually for 2 years in advance of an outbreak. The objective was to assess the impacts of these strategies in not only reducing the outbreak size but also the possibility of complementing them with a reactive strategy close to the outbreak onset. Complementing very low VCs biannually for two years and low reactive VCs is highly effective, e.g. a VC of 10% is predicted to completely avert an outbreak when integrated with a reactive VC of 35%. Annual vaccination is equally effective though at a lower scale. In a large livestock population, averting an outbreak could mean avoiding morbidity and mortality of thousands of animals, reducing vulnerability of local livestock-dependent livelihoods and national economies and, more importantly, reducing chances of virus exposure to humans. Rift Valley fever vectored vaccines are currently being developed [43] and evaluated [44] and this might change (i) the way these vaccines are administered in the field, i.e., some could be given at biannual intervals and or others annual. These combinations can influence the efficacy of the RVFV component of the vaccine. As earlier highlighted in this paper, the shelf-life of current vaccines [12] is shorter than the average inter-outbreak period [14] which presents an economic disincentive to vaccine manufacturers in situations where reactive vaccination campaigns are planned. Similarly, resource-constrained governments are not keen on funding periodic vaccination campaigns partly due to unpredictability of occurrence of the outbreaks. Periodic vaccination campaigns are also a disincentive in situations where livestock population-turn over due to offtakes and expected mortality temporally leads to lower herd immunity. Our analysis is therefore well placed to give policy directions on how vaccination can be used to meet these challenges. Further evaluation of the response impact of integrating periodic and reactive vaccination strategies in preventing the occurrence of a RVF outbreak is an important area for future research and policy development.

Multihost pathogens are more likely to have ecologically different dynamics than pathogens that infect only a single host species. In a host population, multiple host species can be viewed as a form of heterogeneity that partitions the total host population into subpopulations between which the FoI experienced by each host species and the FoI exerted by each host species varies [45]. Based on the assumptions we make in the model, the FoI experienced by cattle is larger due to their higher vector: host ratio arising from their lower number in the population and their assumed larger surface area relative to sheep. The assumptions are qualitatively realistic given the differences in host species’ exposed surface area which is obviously higher in cattle relative to sheep, all other parameters (e.g. blood meal preference) being constant. Similarly, the FoI experienced by vector species from cattle are higher than that from sheep. Consequently, based on our assumptions, the model predicts that cattle dominate the bi-directional RVFV transmission process between hosts and vectors.

With such a pathogen ecological framework where a host species may dominate the virus transmission, we examined the possibility of directing control against either of the host species. Targeting cattle alone provided major protection to cattle and sheep. This benefit arises from the reduction of transmission of RVFV. However, targeting sheep alone provided protection to sheep alone. This prediction, if empirically validated has important policy implications for the implementation of both periodic and reactive vaccination strategies for two reasons: (1) cattle have longer lifespan and lower population turnover relative to sheep and, therefore, would be able to sustain herd immunity for longer, (2) in our case study area (and indeed in all pastoral areas), cattle are fewer relative to sheep (and goats) and (3) in the pastoral communities, cattle are likely to be moved long distances translating to potential spatial spread of RVF compared with sheep and goats. These reasons can greatly influence the cost-effectiveness of a strategy that focuses control against cattle in the population.

Our model, by necessity, includes a number of simplified assumptions about reality in a number of ways that have a bearing on the predictions. We have assumed transmission-related parameters in Table 1 to be similar in both host species. This implies that our outputs were based on two main parameters (i) the use of temporally varying FoI arising from seasonal growth of vectors, and (ii) the different numbers of host species in the population. However, model sensitivity analyses found that RVF cumulative incidence may be influenced most by infectious periods and infectivity in both hosts and vectors (particularly *Culex* spp). Other sensitive parameters include survival and mosquito biting behavior of *Culex* species. The same parameters have been reported to be sensitive to similar outcomes in RVF modelling, e.g. Chitnis et. al [17] reported that an outbreak size was sensitive to vector-to-host ratio, mosquito biting rate and the infectivity of hosts. Other models [15][16][35] reported adequate contact rates between vectors and hosts and the rate of recovery livestock as sensitive to the basic reproduction number. However, their definition of adequate contact rates between vectors and hosts considered a composite term whereas in our model, we disaggregated the term into its individual components including the vector biting rate, host infectivity, blood meal index and vector host ratio. These findings suggest that apart from RVF vaccination, reducing the the probability of transmission from the vector to the host can be effective in RVF outbreak control. In addition, given the importance of understanding RVFV transmission processes, the lack of knowledge about the processes make gathering of relevant field and experimental data on these biological processes an urgent research priority.

Further simplifying assumptions that we make in the analyses of vaccination impacts ignore the individual components that constitute the actual proportion of susceptible hosts vaccinated (herein referred to as an ideal VC (iVC)). This can be obtained as a product of the proportion of vaccinations properly applied (efficiency of vaccination) and the probability that the vaccine would provide protection from infection (the efficacy of the vaccine) [46], both of which limited data are available for RVF. Naturally, these two proportions are each less than 100% in most cases. Multiplicatively, the further the values are from 100%, the less the iVC. A recent study evaluated the effectiveness of RVF Clone 13 vaccine and reported that 67% of vaccinated cattle and between 91% and 97% of vaccinated sheep and goats develop protective antibodies to the vaccine [44]. Applying an efficiency of vaccination of 80% based on the performance of mass vaccination teams as assessed by the Pan African Rinderpest Campaign in pastoral areas in GHA [47], vaccinating an entire population in our study would result to an iVC of approximately 54% in cattle and 76% in sheep. To achieve our simulated VCs, therefore, call for high levels of both the efficiency of vaccination and high efficacy of RVF vaccines such as that reported in sheep and goats [44]. Further explorations required include cost-effectiveness analyses taking account of integrating VCs and time to outbreak, integrating periodic and reactive strategies and directing interventions to one host species under different scenarios of efficiency of vaccination and efficacy of vaccines.

In conclusion, our results suggest that targeted vaccination can be effective in mitigating the impacts of RVF outbreaks. However, it is not always possible to have this measure implemented satisfactorily due to the rapid onset and evolution of RVF epidemics. The analyses further demonstrates that both periodic and reactive vaccination ought to be used strategically to effectively control the disease. In addition, challenges associated with prediction of the outbreak, availability and delivery of vaccines need to be addressed. Factors driving the number of potentially averted cases include the targeted VC and timing of vaccination in relation to the time to the outbreak. Based on our assumptions, cattle appear to dominate RVF transmission between hosts and vectors. Predictably, directing vaccination against cattle, whether in a periodic and/or a reactive vaccination startegy, may be more effective as it confers herd immunity to both species. The work presented here advances our understanding of impacts of different vaccination strategies. We consider that these predictions provide a first step of information needed by policy makers to plan effective periodic and reactive strategies for mitigating the effects of RVF outbreaks. However, detailed cost-benefit analysis should be integrated with these findings to support decision-making and prioritize these strategies.

## Supporting Information

S1 Text Online Supporting Text of Model Equations. (DOCX)
