# Correction: Modelling Vaccination Strategies against Rift Valley Fever in Livestock in Kenya

**DOI:** 10.1371/journal.pntd.0005316

**Published:** 2017-01-26

**Authors:** John M. Gachohi, M. Kariuki Njenga, Philip Kitala, Bernard Bett

Fig 7 is incorrect. The authors have provided a corrected version here.

**Fig 7:**
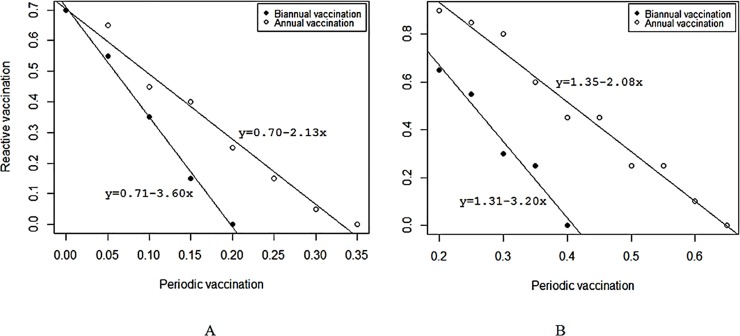
Impacts of integrating various levels of routine and reactive vaccination required to stop an RVF outbreak using a perfect vaccine (Panel A) and imperfect vaccine with 50% vaccine efficacy (Panel B).

## References

[1] Gachohi JM, Njenga MK, Kitala P, Bett B (2016) Modelling Vaccination Strategies against Rift Valley Fever in Livestock in Kenya. PLOS Neglected Tropical Diseases 10(12): e0005049 doi:10.1371/journal.pntd.0005049 27973528 10.1371/journal.pntd.0005049 PMC5156372

