# Supplementary material for: Modelling Vaccination Strategies against Rift Valley Fever in Livestock in Kenya
Source: PLoS Negl Trop Dis. 2016 Dec 14;10(12):e0005049. doi: 10.1371/journal.pntd.0005049 (PMC5156372; doi:10.1371/journal.pntd.0005049)
Supplement: S1 Text — (DOCX) [file pntd.0005049.s001.docx]

**S1 Text. Online Supporting Text of Model Equations**

**Modelling vaccination strategies against Rift Valley fever in livestock in Kenya**

**John M. Gachohi^1,2*^, M. Kariuki Njenga^3^, Philip Kitala^4^, Bernard Bett^2^**

1 School of Public Health, Jomo Kenyatta University of Agriculture and Technology, Kenya, 2

International Livestock Research Institute, Nairobi, Kenya, 3Kenya Medical Research Institute (KEMRI), Nairobi Kenya*,* 4 Faculty of Veterinary Medicine, University of Nairobi, Kenya

***Aedes species population dynamics***

We initiate the simulation with adult *Aedes* mosquitoes laying eggs at each gonotrophic interval. AdE*_(t)_*, SAdL*_(t)_* and SAdP*_(t)_* and SAdA*_(t)_* denote the number of non-infected per life stage, i.e. *Aedes* eggs, *Aedes* larva, *Aedes* pupa and *Aedes* adults respectively. In addition, BIAdE*_(t)_*, IAdL*_(t)_* and IAdP*_(t)_* and IAdA*_(t)_* denote the number infected per life stage, i.e. buried infected *Aedes* eggs, infected *Aedes* larva, infected *Aedes* pupa and infected *Aedes* adults respectively. The first step in the breeding process is the hatching of buried eggs to larvae when conditions become suitable. Hatching of buried *Aedes* eggs uses a fuzzy logic approach. The fuzzy suitability (*f*) of *R$\Sigma$ 21d* is computed by means of a sigmoidal curve as follows:

if *U_1_<* *R$\Sigma$ 21d<S*

(*f*) *R$\Sigma$ 21d* = $1-\cos^{2}\left(\frac{R\sum21d-\cup_{1}}{S-\cup_{1}}\frac{\Pi}{2}\right)$

else, if *S<R$\Sigma$ 21d<*

(*f*) *R$\Sigma$ 21d* = $\cos^{2}\left(\frac{R\sum21d-\cup_{1}}{S-\cup_{1}}\frac{\Pi}{2}\right)$

else

(*f*) *R$\Sigma10d$ =0.* Equation 1

Subsequently, R*21d* is denoted by *pb_(t)_*in the equations. The complete set of the difference equations that execute population dynamics, for either non-infected or infected *Aedes*, via mortality and development to the subsequent life stage (see Table 1 in the main text) between time *t* and *t*+1, is given by:

*Set 1 (Non-infected Aedes species)* Equation 2

*AdE_(t+1)_ = AdE_(t)_+( AdNE*NAedes_(t)_*pb_(t)_*(1-(NAedes_(t)_/AdLCC)))-(AdE_(t)_*0.7)*

*SAdL_(t+1)_ =SAdL_(t)_+(AdE_(t)_*hAd)-(SAdL_(t)_*Adlp)-(SAdL_(t)_*AdLmu*(1-pb_(t)_))*

*SAdP_(t+1)_ =SAdP_(t)_+(SAdL_(t)_*Adlp)-(SAdP_(t)_*Adpa)-(SAdP_(t)_*AdPmu*(1-pb_(t)_))*

*Set 2 (Infected Aedes species)* Equation 3

*IAdL_(t+1)_ =IAdL_(t)_+(BIAdE_(t)_*(hAd*pb_(t)_*(1-(IAdL_(t)_/AdLCC))))-(IAdL_(t)_*Adlp)-(IAdL_(t)_*AdLmu*(1-pb_(t)_))*

*IAdP_(t+1)_ =IAdP_(t)_+(IAdL_(t)_*Adlp)-(IAdP_(t)_*Adpa)-(IAdP_(t)_*AdPmu*(1-pb_(t)_))*

*IAdA_(t+1)_ =IAdA_(t)_ +(IAdP_(t)_*Adpa)-(IAdA_(t)_*AdAmu)*

*Aedes* eggs have high desiccation resistance and can survive dry conditions in a dormant state for months to year, though the actual mortality rate of the buried eggs is unknown. Thus, we insert a density-dependent function that caps the daily number of eggs equal to the initial value. This is done to ensure that the eggs that hatch, on any particular day, are wholly dependent on the value of the fuzzy function.

***Culex species population dynamics***

ClxE*_(t)_*, ClxL*_(t)_*, ClxP*_(t)_*, and ClxA*_(t)_* denote the number of non-infected per life stage, i.e. *Culex* eggs, *Culex* larva, *Culex* pupa and *Culex* adults respectively. The logistic regression model structure was defined as follows:

$\ln\left(\frac{P}{1-P}\right)=\beta_{0}+\beta_{1}x_{1}+\beta_{1}^{2}x_{1}$. Equation 4

These parameters are described in Table 1. The probability estimates (*p*), subsequently denoted *pbclx_(t)_,* used to weight the number of *Culex* eggs that hatch in a day in the simulation model was derived as follows:

**$\frac{e^{\left(\beta_{o}+\beta_{1}x_{1}+\beta_{1}^{2}x_{1}\right)}}{1+e^{\left(\beta_{o}+\beta_{1}x_{1}+\beta_{1}^{2}x_{1}\right)}}$ Equation 5

The complete set of the difference equations (Equation 6) that execute population dynamics of *Culex* species taking account of mortality during each stage and development to the subsequent life stage (see Table 1 in the main text) is given by:

*ClxE_(t+1)_ = ClxE_(t)_+ (ClxNE*ClxA_(t)_*pbclx_(t)_*(1-(ClxL[i]/ClxECC)))-(ClxE_(t)_*clxmue*(1-pbclx_(t)_))-(hClx*ClxE_(t)_)*

*ClxL_(t+1)_ = ClxL_(t)_+ (hClx*ClxE_(t)_)-(Clxlp*ClxL_(t)_)-(ClxLmu*(1-pbclx_(t)_)*ClxL_(t)_)*

*ClxP_(t+1)_ = ClxP_(t)_ + (Clxlp*ClxL_(t)_)-(Clxpa*ClxP_(t)_)-(ClxPmu*(1-pbclx_(t)_)*ClxP_(t)_)*

*ClxA_(t+1)_ = 1+ClxA_(t)_ + (Clxpa*ClxP_(t)_)-(ClxAmu*ClxA_(t)_)*

To prevent uncontrollable population explosion, a density-dependent function was incorporated.

***Integrating host and vector population dynamics and RVFV transmission***

The modelling approach that integrates host age dynamics and virus transmission generates a set of a number of difference equations each with relevant age class and infection states. Events occur successively in the interval [*t*, *t*+1) as follows:

***Set 1 (RVFV transmission to non-infected Aedes)*** Equation 7

*SAdA_(t+1)_ =SAdA[i] +(SAdP_(t)_*Adpa)-(SAdA_(t)_***β_hvA(t)_ -(SAdA_(t)_*AdAmu)*

*EAdA_(t+1)_ =EAdA_(t)_ +(SAdA_(t)_***β_hvA(t_ )-(EAdA_(t)_*AdAmu)*

*HIAdA_(t+1)_ =HIAdA_(t)_+(EAdA_(t)_*LV_(t)_)-(HIAdA_(t)_*AdAmu)*

Where *EAdA* is exposed *Aedes* adults and *HIAdA* are horizontally infected (from infectious hosts) *Aedes* adults.

***Set 2 (RVFV transmission to susceptible Culex)*** Equation 8

*ClxS_(t+1)_ =1+ClxS_(t)_ + (Clxpa*ClxP_(t)_)-(ClxS_(t)_*β_hvC(t)_)- (ClxAmu*ClxS_(t)_)*

*EClxA_(t+1)_ =EClxA_(t)_+(ClxS_(t)_*β_hvC(t)_)-(EClxA_(t)_*LV)-(ClxAmu*EClxA_(t)_)*

*IClxA_(t+1)_ =IClxA_(t)_+(EClxA_(t)_*LV)-(IClxA_(t)_*ClxAmu)*

Where *EClxA* is exposed *Culex* adults and *IClxA* are infected *Culex* adults.

***Set 3 (Age classes and RVFV transmission in cattle)*** Equation 9

Susceptible calves (SC)*_(t+1)_*=SC*_(t)_*+(NC*_(t)_***b_c_**(1-(NC*_(t)_*/CCC)))-(SC*_(t)_***β_vhC_*)-(SC*_(t)_**1/*δc*)-(SC*_(t)_***µC*)

Exposed calves (EC)*_(t+1)_*=EC*_(t)_*+(SC*_(t)_***β_vhC_*)-(EC*_(t)_**1/*δc*)-(EC*_(t)_***µC*)

Infectious calves (IC)*_(t+1)_*=IC*_(t)_*+(EC*_(t)_**1/ε)-(IC*_(t)_**1/*δc*)-(IC*_(t)_***σ_CC_*)-(IC*_(t)_***µC*)

Recovered calves (RC)*_(t+1)_*=RC*_(t)_*+(IC*_(t)_**1/γ)-(RC*_(t)_**1/*δc*)-(RC*_(t)_***µC*)

Susceptible weaners (SW)*_(t+1)_*=SW*_(t)_*+ (SC*_(t)_**1/*δc*)-(SW*_(t)_** *β_vhC_*)-(SW*_(t)_**1/*τc*)-(SW*_(t)_***µC*)

Exposed weaners (EW)*_(t+1)_*=EW*_(t)_*+(SW*_(t)_***β_vhC_*)-(EW*_(t)_**1/*τc*)-(EW*_(t)_***µC*)

Infectious weaners (IW)*_(t+1)_*=IW*_(t)_*+(EW*_(t)_**1/ε)-(IW*_(t)_**1/*τc*) -(IW*_(t)_***σ_AC_*)-(IW*_(t)_***µC*)

Recovered weaners (RW)*_(t+1)_*=RW*_(t)_*+(IW*_(t)_**1/γ)-(RW*_(t)_**1/*τc*)-(RW*_(t)_***µC*)

Susceptible yearlings (SY)*_(t+1)_*=SY*_(t)_*+ (SW*_(t)_**1/*τc*)-(SY*_(t)_** *β_vhC_*)-(SY*_(t)_**1/*ϕc*)-(SY*_(t)_***µC*)

Exposed yearlings (EY)*_(t+1)_*=EY*_(t)_*+(SY*_(t)_***β_vhC_*)-(EY*_(t)_**1/*ϕc*)-(EY*_(t)_***µC*)

Infectious yearlings (IY)*_(t+1)_*=IY*_(t)_*+(EY*_(t)_**1/ε)-(IY*_(t)_**1/*ϕc*)-(IY*_(t)_***σ_AC_*)-(IY*_(t)_***µC*)

Recovered yearlings (RY)*_(t+1)_*=RY*_(t)_*+(IY*_(t)_**1/γ)-(RY*_(t)_**1/*ϕc*)-(RY*_(t)_***µC*)

Susceptible adults (SA)*_(t+1)_*=SA*_(t)_*+ (SY*_(t)_**1/*ϕc*)-(SA*_(t)_** *β_vhC_*)-(SA*_(t)_***µC*)-( SA*_(t)_***Ȱ_c_*)

Exposed adults (EA)*_(t+1)_*=EA*_(t)_*+(SA*_(t)_***β_vhC_*)-(EA*_(t)_***µC*)-(EA*_(t)_***Ȱ_c_*)

Infectious adults (IA)*_(t+1)_*=IA*_(t)_*+(EA*_(t)_**1/ε)-(IA*_(t)_***σ_AC_*) (IA*_(t)_***µC*)- (IA*_(t)_***Ȱ_c_*)

Recovered adults (RA)*_(t+1)_*=RA*_(t)_*+(IA*_(t)_**1/γ)-(RA*_(t)_***µC*)- (RA*_(t)_***Ȱ_c_*)

***Set 4 (Age classes and RVFV transmission in sheep)*** Equation 10

Susceptible lambs (SL)*_(t+1)_*=SL*_(t)_*+(NS*_(t)_***b_S_**(1-(NS*_(t)_*/SCC)))-(SL*_(t)_***β_vhS_*)-(SL*_(t)_**1/*δs*)-(SL*_(t)_***µS*)

Exposed lambs (EL)*_(t+1)_*=EL*_(t)_*+(SL*_(t)_***β_vhS_*)-(EL*_(t)_**1/*δs*)-(EL*_(t)_***µS*) (Equation 44)

Infectious lambs (IL)*_(t+1)_*=IL*_(t)_*+(EL*_(t)_**1/ε)-(IL*_(t)_**1/*δs*) -(IL*_(t)_***σ_LS_*)-(IL*_(t)_***µS*)

Recovered lambs (RL)*_(t+1)_*=RL*_(t)_*+(IL*_(t)_**1/γ)-(RL*_(t)_**1/*δs*)-(RL*_(t)_***µS*)

Susceptible weaners (SW)*_(t+1)_*=SW*_(t)_*+ (SL*_(t)_**1/*δs*)-(SW*_(t)_** *β_vhS_*)-(SW*_(t)_**1/*τs*)-(SW*_(t)_***µS*)

Exposed weaners (EW)*_(t+1)_*=EW*_(t)_*+(SW*_(t)_***β_vhS_*)-(EW*_(t)_**1/*τs*)-(EW*_(t)_***µS*)

Infectious weaners (IW)*_(t+1)_*=IW*_(t)_*+(EW*_(t)_**1/ε)-(IW*_(t)_**1/*τs*) -(IW*_(t)_***σ_AS_*)-(IW*_(t)_***µS*)

Recovered weaners (RW)*_(t+1)_*=RW*_(t)_*+(IW*_(t)_**1/γ)-(RW*_(t)_**1/*τs*)-(RW*_(t)_***µS*)

Susceptible yearlings (SY)*_(t+1)_*=SY*_(t)_*+ (SW*_(t)_**1/*τs*)-(SY*_(t)_** *β_vhS_*)-(SY*_(t)_**1/*ϕs*)-(SY*_(t)_***µS*)

Exposed yearlings (EY)*_(t+1)_*=EY*_(t)_*+(SY*_(t)_***β_vhS_*)-(EY*_(t)_**1/*ϕs*)-(EY*_(t)_***µS*)

Infectious yearlings (IY)*_(t+1)_*=IY*_(t)_*+(EY*_(t)_**1/ε)-(IY*_(t)_**1/*ϕs*) -(IY*_(t)_***σ_AS_*)-(IY*_(t)_***µS*)

Recovered yearlings (RY)*_(t+1)_*=RY*_(t)_*+(IY*_(t)_**1/γ)-(RY*_(t)_**1/*ϕs*)-(RY*_(t)_***µS*)

Susceptible adults (SA)*_(t+1)_*=SA*_(t)_*+ (SY*_(t)_**1/*ϕs*)-(SA*_(t)_** *β_vhS_*)-(SA*_(t)_***µS*)-( SA*_(t)_***Ȱ_s_*)

Exposed adults (EA)*_(t+1)_*=EA*_(t)_*+(SA*_(t)_***β_vhS_*)-(EA*_(t)_***µS*)-(EA*_(t)_***Ȱ_s_*)

Infectious adults (IA)*_(t+1)_*=IA*_(t)_*+(EA*_(t)_**1/ε)-(IA*_(t)_***µS*)- (IA*_(t)_***σ_AS_*) (IA*_(t)_***Ȱ_s_*)

Recovered adults (RA)*_(t+1)_*=RA*_(t)_*+(IA*_(t)_**1/γ)-(RA*_(t)_***µS*)- (RA*_(t)_***Ȱ_s_*)
